# Apport de l’écho-doppler artériel des membres inférieurs dans la prise en charge du pied diabétique à l'hôpital Saint-Jean de Dieu de Thiès (Sénégal)

**DOI:** 10.11604/pamj.2015.22.193.5992

**Published:** 2015-10-27

**Authors:** Aliou Amadou Dia, Désiré Alain Affangla, Jean-Michel Dione, Géraud Akpo, Marie Mbengue, Mamadou Mourtalla Ka, Bernard Marcel Diop

**Affiliations:** 1 Service de Radiologie Hôpital Saint-Jean de Dieu de Thiès, Thiès, Sénégal; 2 Département de Médecine et Spécialités Médicales de l'UFR des Sciences de la Santé Université de Thiès, Thiès, Sénégal; 3 Centre Diabcarmet, Hôpital Saint-Jean de Dieu de Thiès, Thiès, Sénégal

**Keywords:** Pied diabétique, écho-doppler artériel des membres inferieurs, artériopathie diabétique, Diabetic foot, arterial Doppler ultrasound of the lower limb, diabetic arteriopathy

## Abstract

**Introduction:**

Le pied diabétique se définit comme l'ensemble des manifestations trophiques du pied survenant chez le diabétique par atteinte nerveuse, artérielle et ou infectieuse. Le pied diabétique est un problème majeur de santé publique à l’échelle mondiale avec un taux d'amputation de membres inférieurs très élevé. L’écho-doppler artériel des membres inférieurs est de nos jours incontournable dans la prise en charge du pied diabétique. Le but de cette étude est de montrer la place prépondérante qu'occupe l’écho-doppler artériel dans le bilan lésionnel du pied diabétique.

**Méthodes:**

Nous avons mené une étude rétrospective monocentrique incluant 46 patients sur une période de 24 mois, de mars 2012 à mars 2014 à l'hôpital Saint-Jean de Dieu, un des deux hôpitaux de référence de la région de Thiès, doté depuis juillet 2011 d'un centre moderne de traitement du diabète et des maladies cardio-métaboliques (Diabcarmet). Dans les critères d'inclusion, nous avons sélectionné tous les patients diabétiques adressés pour un écho-doppler artériel des membres inférieurs dans le cadre d'une prise en charge du pied diabétique. Etaient exclus de l’étude, les patients artéritiques non-diabétiques et les patients diabétiques asymptomatiques référés pour un bilan écho-doppler de routine.

**Résultats:**

Le sex-ratio était de 1.42 (27 hommes pour 19 femmes). L’âge moyen des patients était de 62,86 ans avec des extrêmes de 23 et 88 ans. 60% des patients (n=28) étaient âgés entre 50 et 70 ans. Le diabète de type 2 était retrouvé chez 95% des patients (n=44) alors que le diabète de type 1 représentait 5% (n=2). La moyenne d’évolution du diabète était estimée à 8 ans, avec des extrêmes de 2 et 20 ans. On notait une atteinte du pied droit chez 24 patients, une atteinte du pied gauche chez 18 patients et une atteinte bilatérale chez 4 patients. La plupart du temps, les lésions du pied diabétique survenaient sur un terrain de diabète déséquilibré (95%). Cliniquement, ces lésions étaient dominées par la gangrène infectieuse du pied (43.47%), l'abolition des pouls tibiaux et pédieux (17.4%), la gangrène infectieuse des orteils (13.07%), la gangrène mixte du pied (4.34%) et le mal perforant plantaire (4.34%). Sur le plan échographique, vingt-six patients ne présentaient aucune anomalie hémodynamique significative, même si sur le plan morphologique la médiacalcose était retrouvée chez tous nos patients (n=46). Les autres lésions morphologiques et hémodynamiques artérielles étaient dominées par la sténose serrée de l'artère fémorale superficielle chez 6 patients soit 13.04%, les sténoses des artères tibiales antérieures et postérieures chez 4 patients (6.52%) et l'association de plusieurs lésions artérielles chez 4 patients (8.7%). Le taux d'amputation, dans notre série, était de 21.7%.

**Conclusion:**

Le pied diabétique est une complication potentiellement grave du diabète, en Afrique sub-saharienne du fait d'un fort taux d'amputation de membre. L’écho-doppler artériel des membres inférieurs est un moyen d'imagerie non irradiant et non invasif indispensable dans la prise de décision thérapeutique du pied diabétique.

## Introduction

Le pied diabétique est un problème majeur de santé publique à l’échelle mondiale avec un taux d'amputation de membres inférieurs très élevé et des conséquences souvent dramatiques sur le plan social, psychologique et économique. Les complications podologiques du diabète sont dominées par la neuropathie diabétique, l'artériopathie diabétique et l'infection des ulcérations du pied [1, 2]. Certaines études rétrospectives évaluent l'incidence des plaies chroniques chez les diabétiques à 2 à 3 ulcères/100 patient /an [3, 4]. En Afrique, les ulcères et les amputations du pied sont malheureusement très courants. Souvent, la pauvreté, le manque d'hygiène et la marche à pieds nus interagissent pour aggraver l'impact des lésions du pied causées par le diabète [5, 6]. 5 à 10% des patients diabétiques auront un jour à subir une amputation mineure ou majeure d'un membre inférieur. Le diabète est à l'origine d'environ 40 à 60% des amputations non traumatiques et 85% de ces amputations sont précédées par une ulcération du pied. Le risque d'amputation au niveau des membres inférieurs est 15 à 30 fois plus élevé dans la population diabétique que dans la population générale [3, 4, 7, 8]. L’écho-doppler artériel, avec cartographie précise des axes artériels de jambe et de pied, est devenu non seulement l'examen de première intention dans le diagnostic de l'artériopathie diabétique des membres inférieurs mais également un outil incontournable dans la prise de décision thérapeutique. Le Doppler artériel est un examen d'une totale innocuité, non irradiant, accessible et reproductible mais opérateur-dépendant qui permet une analyse morphologique et hémodynamique satisfaisante des différents axes artériels [9–11]. Le but de cette étude est de montrer la place prépondérante qu'occupe l’écho-doppler artériel dans le bilan lésionnel du pied diabétique.

## Méthodes

### Patients

Nous avons mené une étude rétrospective monocentrique sur 24 mois, de mars 2012 à mars 2014, incluant 46 patients hospitalisés pour pied diabétique. Dans les critères d'inclusion, nous avons sélectionnés tous les patients diabétiques adressés pour un écho-doppler artériel des membres inférieurs dans le cadre d’‘une prise en charge du pied diabétique. Etaient exclus de l’étude, les patients artéritiques non diabétiques et les patients diabétiques asymptomatiques référés pour un bilan echo-doppler de routine.

### Cadre de l’étude

L’étude a été menée à l'hôpital Saint-Jean de Dieu, un des deux hôpitaux de référence de la région de Thiès. Tous nos patients étaient référés par le centre Diabcarmet qui est une structure pluridisciplinaire de l'hôpital Saint-Jean de Thiès traitant le diabète et les maladies cardio-métaboliques. Depuis son inauguration en juillet 2011, le centre Diabcarmet permet non seulement de désengorger le centre diabétique Marc Sankalé de Dakar qui jusque-là était la seule structure hospitalière sénégalaise spécialisée dans la prise en charge du diabète mais également de traiter sur place les patients de la région de Thiès qui étaient obligés de se rendre jusqu’‘a Dakar, ce qui augmentait le coût financier de leur prise en charge, dans un contexte socio-économique difficile.

### Protocole d'examen de l’écho-doppler

Tous nos patients avaient été explorés avec un appareil d’échographie-Doppler couleur de marque Mindray DC-7 ^®^ qui permettait la sauvegarde de toutes les données des patients ainsi que la conversion des images en format JPEG grâce à un logiciel DICOM. L'examen écho-doppler était réalisé en suivant l'anatomie du réseau artériel depuis l'aorte abdominale jusqu'aux artères pédieuses et comprenait essentiellement des coupes longitudinales. Ainsi, on procédait à une analyse méthodique et rigoureuse des spectres Doppler et de la morphologie de l'aorte abdominale, des axes iliaques, de la bifurcation fémorale commune, des axes fémoro-poplités, de la bifurcation poplitée et du lit d'aval jambier. L’étage aorto-iliaque était exploré chez un patient en décubitus dorsal avec une sonde sectorielle de 3Mhz; quant aux axes fémoro-poplités et artériels distaux, l'exploration était faite de façon symétrique, avec une sonde linéaire superficielle de 7Mhz, chez un patient en décubitus dorsal ou latéral, jambes semi-fléchies pour l’étude du réseau artériel postérieur. On s'aidait du mode B pour l'appréciation de la paroi artérielle, de la médiacalcose et pour la caractérisation des plaques athéromateuses. Le module couleur était utilisé pour apprécier le remplissage vasculaire, les phénomènes de turbulences (aliasing) sur les sites de sténoses, mais également pour le repérage vasculaire en distalité du fait de l'aspect grêle des artères.

### Saisie et analyse des données

Toutes les informations (âge, sexe, indication clinique, latéralité des lésions, type de diabète, durée d’évolution, état d’équilibre du diabète, résultats de l'examen écho-doppler, aspects post-thérapeutiques) étaient répertoriées sur un fichier Excel 2007. Le calcul des moyennes, des standard déviations, des extrêmes, et des pourcentages était réalisé grâce au logiciel IBM SPSS statistics 21^®^.

### Limites de l’étude

Les limites de l’étude sont liées au caractère monocentrique. Nous nous sommes plus focalisés sur l'analyse morphologique et hémodynamique des lésions artérielles que sur l’étude des index de Pression systolique (IPS) car d'une part, tous nos patients étaient porteurs d'une médiacalcose qui rendait les chiffres obtenus non fiables, d'autre part la majeure partie de nos patients avaient une gangrène humide au niveau de la cheville ou des orteils qui rendait impossible la pose d'un brassard. Les données thérapeutiques étaient manquantes dans certains dossiers de patients car ces derniers avaient été transférés vers d'autres structures hospitalières.

## Résultats

**Figure 1:**
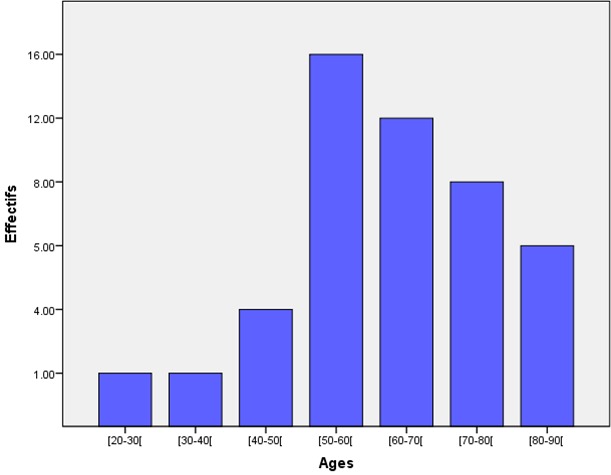
Répartition des patients par tranches d’âge

**Figure 2:**
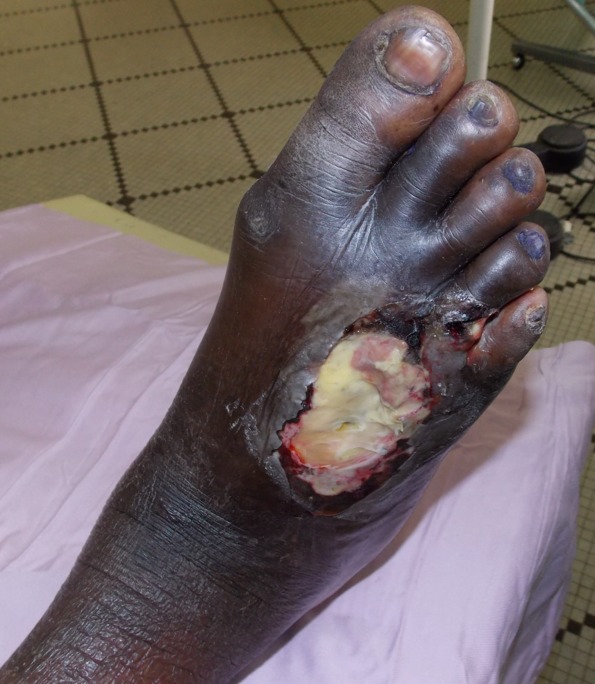
Gangrène infectieuse de l'avant-pied droit chez un diabétique

**Figure 3:**
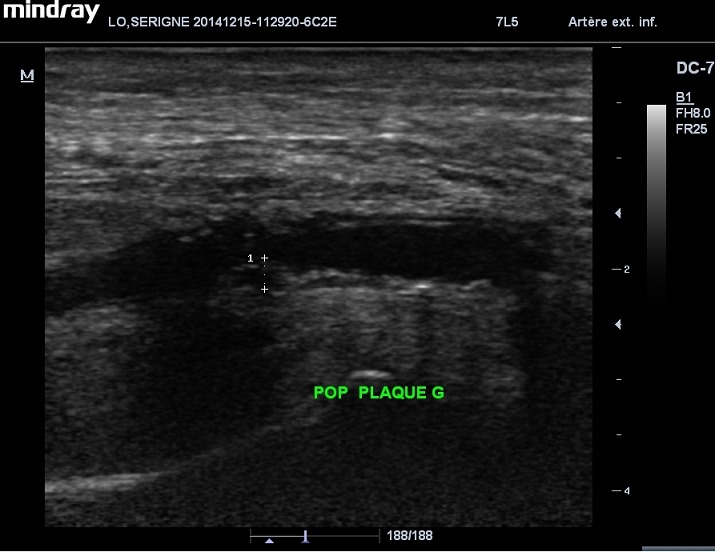
Echographie en mode B montrant une plaque athéromateuse de l'artère poplitée gauche responsable d'une sténose non serrée associée à une médiacalcose diffuse (calcifications de la média)

**Figure 4:**
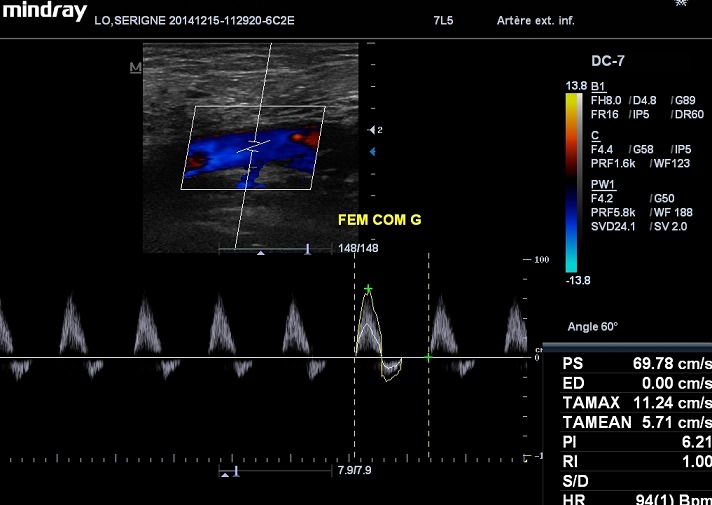
Spectre Doppler d'une sténose non serrée (‘75%) d'une artère fémorale commune avec une accélération modérée des vitesses systoliques et aliasing

**Figure 5:**
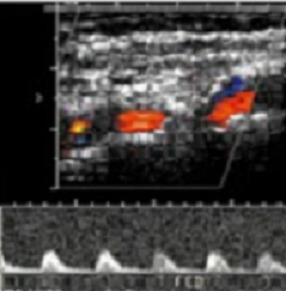
Spectre Doppler objectivant une démodulation du flux en aval d'une sténose serrée ‘75% d'une artère tibiale antérieure

Le sex-ratio était de 1.42 (27 hommes pour 19 femmes). L’âge moyen des 46 patients était de 62,86 ans avec des extrêmes de 23 et 88 ans. 60% des patients (n=28) étaient âgés entre 50 et 70 ans (Figure 1). Le diabète de type 2 était retrouvé chez 95% des patients (n=44) alors que le diabète de type 1 représentait 5% (n=2). La moyenne d’évolution du diabète était estimée à 8 ans, avec des extrêmes de 2 et 20 ans. On notait une atteinte du pied droit chez 24 patients, une atteinte du pied gauche chez 18 patients et une atteinte bilatérale chez 4 patients. Les lésions du pied diabétique survenaient la plupart du temps, sur un terrain de diabète déséquilibré (95%). Cliniquement, ces lésions étaient dominées par la gangrène infectieuse du pied (43.47%) (Figure 2), l'abolition des pouls tibiaux et pédieux (17.4%), la gangrène infectieuse des orteils (13.07%), la gangrène mixte du pied (4.34%) et le mal perforant plantaire (4.34%) (Tableau 1). Sur le plan échographique, vingt-six de nos patients (56.5%) ne présentaient pas d'anomalie hémodynamique significative même si sur le plan morphologique, on pouvait noter la médiacalcose chez tous nos patients (Figure 3). Les autres lésions morphologiques et hémodynamiques artérielles étaient dominées par la sténose serrée de l'artère fémorale superficielle chez 6 patients soit 13.04%, les sténoses des artères tibiales antérieure et postérieure chez 4 patients (6.52%) et l'association de plusieurs lésions artérielles chez 4 patients (8.7%) (Tableau 2). Le diagnostic de sténose serrée reposait sur des signes directs (accélération des vitesses systoliques > 2m/secondes, phénomènes de turbulences au niveau du site de sténose avec aliasing) et sur un frein hémodynamique d'aval (amortissement ou démodulation des flux) (Figure 4 et Figure 5). Le diagnostic de thrombose ou occlusion artérielle était posé devant l'absence de signal Doppler couleur ou énergie, la présence de matériel intraluminal en échographie B et un tracé plat en mode pulse. Sur le plan thérapeutique, nous avons noté une amputation mineure (désarticulation d'un orteil, amputation de Chopart ou Lisfranc) chez 6 patients et une amputation majeure (amputation de jambe ou de cuisse) chez 4 patients, soit un pourcentage total de 21.7%.

**Tableau 1:** Indications cliniques de l’écho-doppler artériel

Indications	Effectifs (n)	Pourcentage (%)
Abolition des pouls pédieux et tibiaux	8	17.39
Gangrène infectieuse du pied	20	43.47
Gangrène ischémique du pied	1	2.17
Gangrène mixte du pied	2	4.34
Mal perforant plantaire	2	4.34
Nécrose infectieuse des orteils	6	13.04
Nécrose ischémique des orteils	1	2.17
Nécrose mixte des orteils	2	4.34
Oedème du pied	3	6.52
Phlegmon de la jambe	1	2.17

**Tableau 2:** Résultats de l’écho-doppler artériel des membres inférieurs

Résultats de l’écho-doppler artériel	Effectifs (n)	Pourcentage (%)
Absence d'anomalie hémodynamique significative	26	56.52
Médiacalcose	46	100
Plaques athéromateuses fémorales sans sténose hémodynamique	2	4.34
Sténose serrée de l'artère fémorale superficielle	6	13.04
Occlusion de l'artère fémorale superficielle	1	2.17
Sténose de l'artère poplitée	1	2,17
Occlusion de l'artère poplitée	2	4.34
Sténose des artères tibiales antérieures et postérieures	3	6.52
Occlusion de l'artère pédieuse dorsale	1	2.17
Association de plusieurs lésions artérielles	4	8.7

## Discussion

Les troubles trophiques du pied chez le diabétique sont la conséquence de plusieurs mécanismes physiopathologiques que sont la neuropathie, l'artériopathie et l'infection. La neuropathie périphérique, sensitivomotrice et autonome, est fréquente et représente la principale complication à l'origine des lésions du pied diabétique, avec perte d'alerte douloureuse, déformations du pied, hyper appui et sécheresse cutanée. L'artériopathie, plus fréquente et plus grave que chez le patient non diabétique, est un facteur d'aggravation très important responsable de retard de cicatrisation et de gangrène à l'origine fréquente d'amputation. L'infection est aussi un facteur d'aggravation majeur par son risque d'extension profonde, notamment vers l'os qui peut conduire à l'amputation, et d'extension générale avec son risque vital. L'artériopathie diabétique des membres inférieurs est le plus souvent asymptomatique, notamment la claudication intermittente est absente chez plus de 50% des diabétiques; il y a moins de différence entre les sexes. L'artérite touche deux hommes diabétiques pour une femme diabétique, alors que chez le non-diabétique le rapport est de dix hommes pour une femme. Dans notre série, nous avons retrouvé une prédominance masculine avec un sex-ratio à 1.42. Les diabétiques développent trois types de lésions vasculaires périphériques: la microangiopathie, l'artériosclérose et l'athérosclérose. La microangiopathie est une complication quasi-spécifique du diabète, ayant pour facteur causal unique l'hyperglycémie. Bien que son rôle soit établi dans la genèse des lésions rénales et oculaires, la microangiopathie n'est jamais responsable par elle-même d'une nécrose distale d'orteil qui est toujours secondaire à une lésion des artères musculaires même s'il s'agit d'artères distales du pied; l'artériosclérose est le deuxième type de lésion vasculaire, caractérisée par une hyperplasie intimale et une dégénérescence hyaline de la média avec dépôts de substances muccopolysaccharidiques. L’évolution de telles lésions se fait vers la sclérose et la médiacalcose. Cette médiacalcose, souvent associée à la neuropathie, est une calcification de la média des artères très caractéristique du diabète. Elle rigidifie la paroi artérielle mais n'est pas synonyme d'obstruction artérielle, ni d'ischémie; l'athérosclérose est la véritable lésion artérielle responsable de l'ischémie et du pronostic de cicatrisation des ulcérations [1–7].

L'analyse de nos données épidémiologiques nous montre que le pied diabétique est surtout l'apanage des sujets âgés entre 50 et 70 ans, avec une moyenne d’âge de 62.5 ans. Cette moyenne d’âge est supérieure à celle de Monabeka qui avait retrouvé une valeur de 54.6 ans [8]. La durée moyenne d’évolution du diabète, chez nos patients était de 8 ans, chiffre proche de celui d'Ekpebegh qui était de 8.3 ans [9]. On pouvait noter une prédominance de l'atteinte du pied droit (45% des patients), bien que nous n'ayons pas retrouvé de séries traitant la latéralité des lésions. Dans notre série, les indications cliniques étaient dominées par la gangrène infectieuse du pied et des orteils (56.5%). Ces données étaient en parfaite corrélation avec les études africaines, bien que nos chiffres soient inférieurs aux séries de Monabeka et de Kouamé [8, 10]. La fréquence des lésions infectieuses peut-être expliquée par le non-respect des règles hygiéno-diététiques et la non-observance du traitement, le tout souvent dans un contexte socio-économique difficile [6, 9]. Dans la région de Thiès, il n'est pas rare que le pied diabétique soit le motif de découverte du diabète, surtout chez les patients en provenance des zones rurales, ou le taux d'analphabétisation reste encore élevé. L´e'cho-doppler est aujourd´hui une méthode de choix dans le diagnostic et l´évaluation de l´artériopathie chronique oblitérante des membres inférieurs, particulièrement chez le diabétique. Non-invasive, peu coûteuse, fiable, reproductible mais opérateur-machine et patient-dépendante, cette méthode permet de réaliser une cartographie anatomique très précise des axes artériels des membres inférieurs. De plus, elle nous renseigne sur le retentissement fonctionnel de l´artériopathie et guide au mieux le choix de la thérapeutique et la surveillance des traitements. L’écho-doppler a des performances comparables à celles de l'artériographie, avec une sensibilité pour la détection des occlusions et des sténoses estimée respectivement à 95% et 92% et des spécificités respectives de l'ordre de 99% et 97% [10–18]. Dans notre série, l’écho-doppler ne retrouvait pas d'anomalie hémodynamique chez 26 patients (56.5%). Les lésions du pied diabétique de ces patients correspondaient essentiellement à des lésions infectieuses et ont pu bénéficier d'un traitement médical et de pansements locaux. Par contre, on notait des anomalies morphologiques et hémodynamiques chez les 20 patients restants (43,5%). La médiacalcose était présente chez la totalité de nos patients (n=46), un chiffre supérieur à la série de Kouamé qui retrouvait 4 cas de médiacalcose sur une population de 70 patients [10].

La médiacalcose de Monckeberg se définit comme étant des calcifications de la média (particulièrement au niveau des couches limitantes élastiques) sont particulièrement fréquentes et abondantes chez le diabétique. Ces calcifications rendent la paroi artérielle rigide, réduisant sa compliance mais posant aussi des problèmes pratiques dans la mesure de l'indice de pression systolique, qui se trouve élevé ou faussement normal, même en cas d'artériopathie obstructive. Six patients (13.04%) présentaient une sténose de l'artère fémorale superficielle. L'artère fémorale est le plus souvent le siège de surcharge athéromateuse que de sténose. Dans notre étude, l’écho-doppler a pu ainsi détecter les sténoses et les occlusions artérielles et a permis d'apprécier le caractère hémodynamiquement significatif ou non d'une sténose de même que le retentissement sur le lit artériel d'aval. Une association de plusieurs lésions artérielles étaient notée chez 4 patients (8.7%), ceci pouvant être expliqué par le caractère multi-segmentaire de l'artériopathie diabétique. L'atteinte des artères distales est expliquée, d'une part, par la microangiopathie qui est responsable d'une dégradation progressive de l'amplitude et de la modulation des tracés Doppler [17]. Dans notre structure hospitalière, le traitement des lésions sévères du pied diabétique reste malheureusement dominé par les amputations qui peuvent être mineures (désarticulation d'un orteil, amputation de Chopart ou Lisfranc) ou majeures (amputation de jambe ou de cuisse). Nous avons noté une amputation mineure chez 6 patients et majeure chez 4 patients, soit un pourcentage total de 21.7%. Ce chiffre ne reflète peut-être pas la réalité, bien qu'inférieur à celui de Monakeba qui retrouvait un taux d'amputation de 42.2% [8], car certains de nos patients ayant été transférés vers d'autres structures hospitalières ont pu y bénéficier d'une amputation.

## Conclusion

Le pied diabétique est l'une des complications les plus sévères du diabète, en Afrique Sub-saharienne avec une morbi-mortalité élevée liée à un fort taux d'amputation de membres. La prévention des lésions du pied diabétique doit obligatoirement passer par une sensibilisation sur l'observance des mesures hygiéno-diététiques. Dans nos conditions d'exercice où l'accès à la chirurgie de revascularisation du membre inférieur reste difficile et onéreux, l’écho-doppler artériel s'impose avant même l'apparition des troubles trophiques chez le diabétique, afin de réduire le taux d'amputation des membres.

## References

[1] Ha Van G (2008). Artériopathie diabétique des membres inférieurs. Le pied diabétique. Abrégé.

[2] Ha Van G (2014). Le pied diabétique. Revue du rhumatisme monographies.

[3] International Working Group on the Diabetic Foot, IWGDF http://www.sfdiabete.org/sites/sfd…/Recommandations_IWGDF_2011.

[4] Malgrange D (2008). Physiopathologie du pied diabétique. La Revue de Médecine Interne..

[5] Boulton A (2005). Le pied diabétique: épidémiologie, facteurs de risque et état des soins. Diabetes Voice..

[6] McLarty DG, Pollitt C, Swai ABM (1990). Diabetes in Africa. Diabetic Medicine.

[7] Pierret C, Tourtier JP, Bordier L, Blin E, Duverger V (2011). Revascularisation du pied diabétique. La Presse Médicale..

[8] Monabeka HG, Nsakala-Kibangou N (2001). Aspects épidémiologiques et cliniques du pied diabétique au CHU de Brazzaville. Bull Soc Pathol Exot..

[9] Ekpebegh CO, Iwuala SO, Fasanmade OA (2009). Diabetes foot ulceration in a Nigerian Hospital: in-hospital mortality in relation to the presenting demographic, clinical and laboratory features. Int Wound J..

[10] Kouamé N, Koffi D, N'goan-Domoua AM (2011). L’échographie doppler dans la prévention des amputations des membres inférieurs du diabétique en Côte d’Ivoire. Médecine Nucléaire..

[11] Fawzi SF, Alex DA (2007). L’écho-doppler modifie rarement les décisions dans la prise en charge de l'ischémie chronique des membres inférieurs. Annales de Chirurgie Vasculaire..

[12] Becker F, Luizy F, Baud JM, Pichot O (2011). Standards de qualité pour la pratique des examens Doppler et écho-Doppler artériel des membres inférieurs en médecine vasculaire:rapport de la Société française de médecine vasculaire (SFMV). Journal des Maladies Vasculaires..

[13] Laissy JP, Pernes JM (2004). Quand, comment et pourquoi réaliser une imagerie des artères des membres inférieurs?. Journal de Radiologie..

[14] Consensus online. Sténose artérielle des membres inférieurs http://www.consensus-online.fr/Stenoses-arterielles-des-membres.

[15] Hodgkiss-Harlow KD, Bandyk DF (2013). Interpretation of arterial duplex testing of lower-extremity arteries and interventions. Semin Vasc Surg..

[16] Got I (2008). Artériopathie et pied diabétique. La Revue de Médecine Interne.

[17] Benhamou AC, Dadon M, Emmerich J, Fontaine P, Got I, Guillausseau PJ (1997). Artériopathie des membres inférieurs chez le diabétique. Diabetes Métab..

[18] Fredenrich A, Bouillanne PJ, Batt M (2004). Artériopathie diabétique des membres inférieurs. EMC-Endocrinologie..

